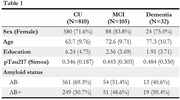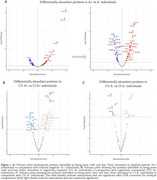# Characterizing blood‐based biomarkers of AD pathology using both SIMOA and NULISA technologies among Indigenous Africans: Pioneering experience with the VALIANT Cohort

**DOI:** 10.1002/alz70856_103752

**Published:** 2025-12-25

**Authors:** Tolulope O Akinyemi

**Affiliations:** ^1^ Lead City University, Ibadan, Oyo, Nigeria

## Abstract

**Background:**

Biofluid biomarkers in Alzheimer's disease and related dementia (ADRD) research is in infancy in Africa. Most existing data come from populations in the Global North, where risk factors and comorbidities differ from those in the Global South. Therefore, biomarker studies in diverse populations are essential to expand and validate their clinical utility. As such, we investigated the expression of plasma biomarkers in a large community‐based cohort of older Nigerians, utilizing two cutting‐edge multiplex technologies: Single molecule array (SIMOA) technology and NUcleic acid Linked Immuno‐Sandwich Assay (NULISA).

**Method:**

We included in this study 947 older Nigerian Africans (65 yr ± 10.4; 73% women) of Yoruba ethnicity (97%) participating in the VALIANT cohort (Table 1). Together with clinical diagnosis, these individuals had plasma samples analyzed using the SIMOA and the NULISAseq (CNS panel) platforms for AD biomarkers plasma (Aβ40, Aβ42, NfL, GFAP and *p*‐Tau 217) and novel protein signatures. Individuals were further categorized according to their amyloid (Aβ) status, in positive (+) or negative (‐) using SIMOA plasma pTau217. Linear models, adjusted by age and sex, evaluated differentially abundant proteins (DAPs) contrasting the Aβ groups as well as cognitive status (CU vs CI) within Aβ+ and Aβ groups.

**Result:**

Plasma *p*‐tau217, GFAP and NfL demonstrated significant progressive increment across groups, while Aβ42/Aβ40 showed higher ratio in MCI than CU and dementia groups, with similar results across platforms. Linear models revealed over 50 differentially abundant proteins (DAP) with increased or decreased expression between Aβ+ and Aβ‐ groups (Figure 1). When comparing cognitive status within Aβ+ individuals, GFAP, POSTN and IGFBP7 showed higher abundance in cognitively impaired individuals.

**Conclusion:**

Preliminary results show DAPs related with inflammation, neurodegeneration and amyloid and tau pathologies in the presence of Aβ pathology, confirming previous data. Ongoing analysis will further explore the data to reveal singularities and similarities with current knowledge.